# Biomimetic Stratum Corneum Liposome Models: Lamellar Organization and Permeability Studies

**DOI:** 10.3390/membranes13020135

**Published:** 2023-01-20

**Authors:** Susmita Roy, James C. S. Ho, Douglas L. C. Teo, Shikhar Gupta, Madhavan Nallani

**Affiliations:** 1 Center for Biomimetic Sensor Science, School of Materials Science and Engineering, Nanyang Technological University, Singapore 637553, Singapore; 2 Procter & Gamble International Operations SA SG Branch, Singapore 138547, Singapore; 3 ACM Biolabs Pte Ltd., Singapore 638075, Singapore

**Keywords:** stratum corneum, ceramide, free fatty acid, cholesterol, biophysical characterization, lamellar organization, permeability

## Abstract

The stratum corneum (SC), the outer layer of the skin, plays a crucial role as a barrier protecting the underlying cells from external stress. The SC comprises three key components: ceramide (CER), free fatty acid (FFA), and cholesterol, along with small fractions of cholesterol sulfate and cholesterol ester. In order to gain a deeper understanding about the interdependence of the two major components, CER and FFA, on the organizational, structural, and functional properties of the SC layer, a library of SC lipid liposome (SCLL) models was developed by mixing CER (phytosphingosine or sphingosine), FFA (oleic acid, palmitic acid, or stearic acid), cholesterol, and cholesterol sulfate. Self-assembly of the SC lipids into lamellar phases was first confirmed by small-angle X-ray scattering. Short periodicity and long periodicity phases were identified for SCLLs containing phytosphingosines and sphingosine CERs, respectively. Furthermore, unsaturation in the CER acyl and FFA chains reduced the lipid conformational ordering and packing density of the liposomal bilayer, which were measured by differential scanning calorimetry and Fourier transform infrared spectroscopy. The introduction of unsaturation in the CER and/or FFA chains also impacted the lamellar integrity and permeability. This extensive library of SCLL models exhibiting physiologically relevant lamellar phases with defined structural and functional properties may potentially be used as a model system for screening pharmaceuticals or cosmetic agents.

## 1. Introduction

**Scheme 1:**
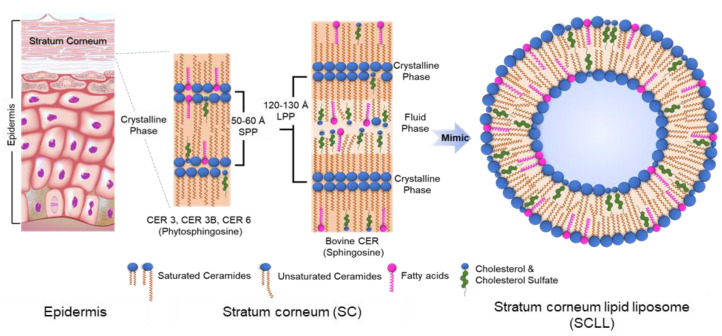
The stratum corneum (SC) and SC lipid liposome (SCLL) lipid organization. SC lipids are organized into short periodicity phase (SPP) and long periodicity phase (LPP). The model (right) represents a proposed SC lipid liposome (SCLL) model.

The stratum corneum (SC) is the outermost layer of the human skin. It functions as a physical barrier that protects the underlying cells from chemical and mechanical stresses as well as microbial penetration. The SC is primarily composed of flattened dead cells, corneocytes, and an extracellular milieu of lipids (Scheme 1). These lipids help to prevent the excessive loss of water from the skin and to regulate the absorption of drugs administered topically and transdermally [1]. The key lipid constituents in the SC are ceramide (CER), free fatty acid (FFA), and cholesterol, along with small amounts of cholesterol sulfate and cholesterol ester [2]. Imbalance in their relative abundance or chemical configurations of the lipid tail and head groups implicates the structural integrity of the lamellar phase and is associated with skin disorders [3]. In addition to its structural role, CERs are bioactive sphingolipids that mediate intracellular signal transduction homeostasis, involving cell cycle, cell differentiation, as well as cell death and survival [3].

The molecular organization of a typical SC layer is not fully resolved. A widely referred model describes the organization into two lamellar phases with repeat distances of approximately 120–130 Å and 50–60 Å, referred to as the long periodicity phase (LPP) and short periodicity phase (SPP), respectively [4,5,6,7,8,9] (Scheme 1). The LPP consists of a sandwich structure with three zones stacked normal to the membrane plane—a fluid middle sublattice with a narrow central lipid lamina and two adjacent regions with crystalline layers [10]. The SPP possesses one lipid bilayer consisting of a more condensed structure, where the distance between the termini head groups is reduced [10]. Other models describe the SC layer as alternating phases with repeating units of 45 Å and 65 Å [11,12] or a multilamellar layer with ~50 Å [13] or ~55 Å repeat [14]. The latter two studies are based on cryogenic electron microscopy analysis, and the LPP was not observed [13,14], suggesting a dominant role of SPP. The CERs belong to a family of sphingolipids that consist of long fatty acid hydrocarbon chains (saturated or unsaturated) and a sphingoid moiety. CERs are subclassified according to the constituent sphingoid variants, including sphingosine (S), trihydroxylated phytosphingosine (P), 6-hydroxysphingosine, or dihydrosphingosine, while the fatty acid moiety can either be nonhydroxylated (N), α-hydroxylated (A), or esterified ω-hydroxylated (EO), with chain lengths ranging from 18 to 26 carbon atoms [15,16,17]. Notably, EOS has been associated with the LPP formation, and its absence or a reduced abundance contributes to disease states [10,18].

The literature on the impact and interactions of different SC lipids, from both experiments, relying on small-angle X-ray scattering (SAXS), fluorescence spectroscopy, differential scanning calorimetry (DSC), and Fourier-transform infrared (FTIR) spectroscopy [4,5,19,20,21,22,23], as well as theoretical approach [24,25], is rich and extensive. The permeability of the SC membrane is highly dependent on the CER chain length [19] and the polarity of its head groups [20,26,27]. Furthermore, model membranes based on synthetic CERs suggest that CER chain length (long-chain (with 16–18 carbons) versus very long-chain (with more than 20 carbons)), unsaturation degree, and headgroup architecture influence the lamellar organization [23,28,29,30,31,32,33,34,35,36,37,38,39,40,41]. The second major component in SC, the FFAs, characterized by their acyl chain length and degree of unsaturation, also impart physical effects on the overall structural organization, such as membrane permeability, acyl chain packing, and lateral pressure profile [42,43,44,45]. Importantly, the effects of long-chain saturated fatty acids on SC lipids do not necessarily mirror that of phospholipid bilayers [40], which emphasizes the need of empirical measurement for modified lipid compositions.

**Figure 1:**
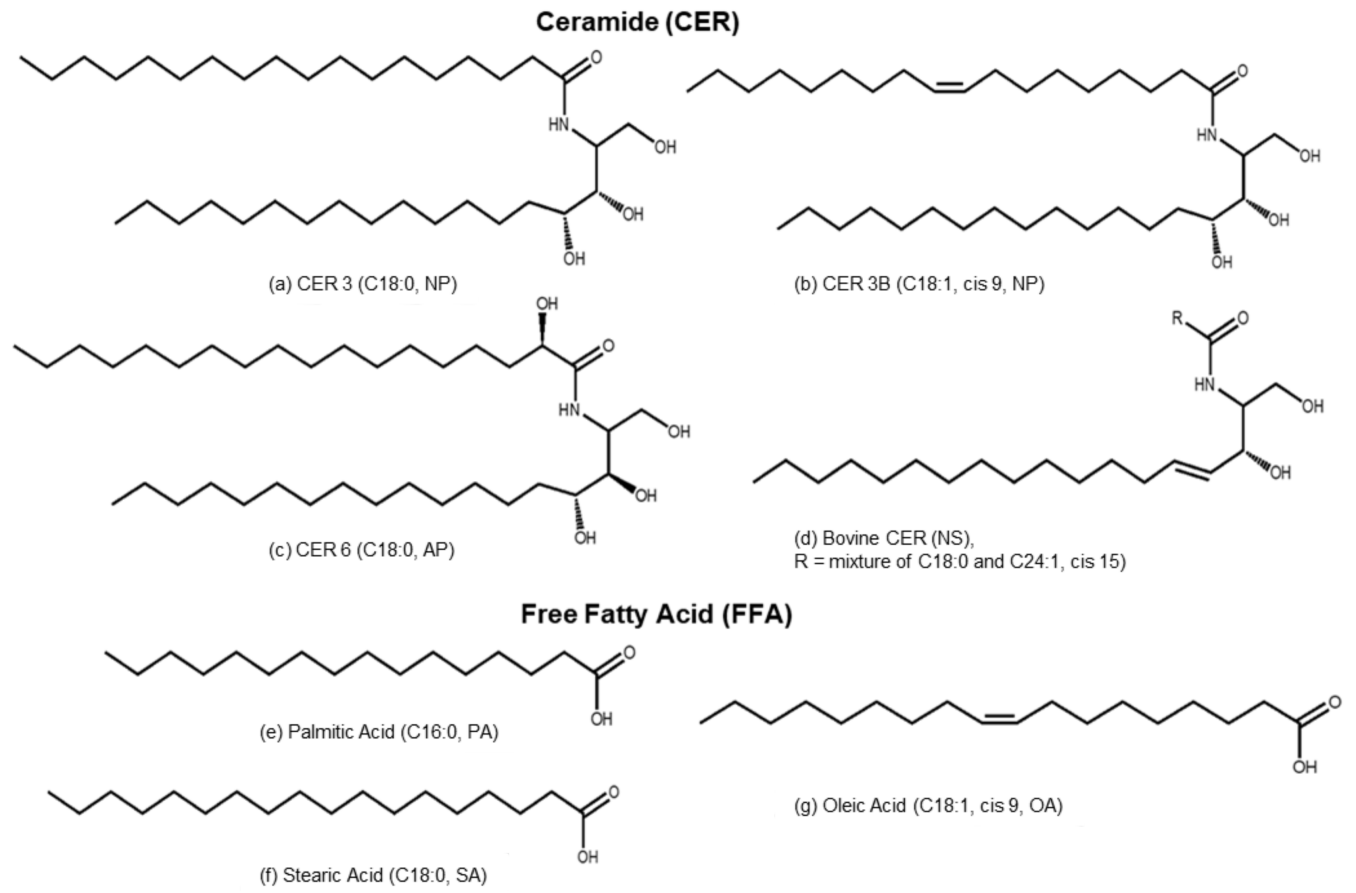
The chemical structures of the ceramides (CERs) and free fatty acids (FFAs) used. NP = CERs consisting of a nonhydroxylated (N) fatty acid moiety and a trihydroxylated phytophingosine (P) moiety. Two types of CER (NP), decorated with C18:0 or C18:1 fatty acids, are studied. AP = CERs consisting of an α-hydroxylated (A) fatty acid moiety and a trihydroxylated phytophingosine (P) moiety. NS = CERs consisting of a nonhydroxylated (N) fatty acid moiety and a sphingosine (S) moiety. The bovine CER contains a mixture consisting of C18:0 or C24:1 as the fatty acid moiety.

In this study, we aim to examine a panel of SC lipid liposome (SCLL) models focusing on using structurally similar CERs and FFAs, rather than the physiologically complex mixture, to determine the structural–function relationship and the interdependence of the two major SC lipid components. We surmised that a well-characterized structure-function relationship obtained from side-by-side comparison of similar SCLL models, focusing on SPP and represented in the same study, is valuable as a toolbox to understand the effect of novel membrane-active compounds. We vary chain length, degree of unsaturation, and head group architecture of the CERs and FFAs and assess the sensitivity of the SCLL models when challenged with membrane perturbing agent (Triton X-100). The composition we used for all the SCLLs in this study is based on an established SCLL model with a mixture of CERs (40%), FFA (25%), cholesterol (25%), and cholesteryl sulfate (10%) [46,47,48,49,50]. This lipid composition forms large unilamellar liposomes that are known to be structurally stable at physiological pH [48,49,50]. Specifically, 12 SCLLs with different combinations of CERs and FFAs were produced. Four CERs represented by three synthetic phytosphingosine ceramides (CER 3, CER 3B, and CER 6) and one sphingosine-ceramide (bovine CER), along with three FFAs represented by saturated C16:0 (palmitic acid, PA) and C18:0 (stearic acid, SA) and monounsaturated C18:1 cis 9 (oleic acid, OA), were studied for their relative influence on SCLL lamellar organization and conformational ordering, permeability, and thermotropic behavior. CER 3 and CER 6 have a saturated stearic acid side chain, whereas CER 3B contains an oleic acid moiety. The bovine CER consists of a mixture of stearic acid and nervonic acid (C24:1 cis 15) moieties (Figure 1).

We benchmarked and rationalized our SCLL models based on known spectroscopic and calorimetric signatures and showed that the structural properties of these SCLL models are consistent with earlier works by the others [33,34,35,36,37,51]. The current study also provides a detailed picture on the molecular phase behavior, lamellar arrangement, and their correlation with membrane permeability readout. We find SCLLs composed of phytosphingosine CERs with C18 acyl chain form SPP, whereas the characteristic LPP is exclusively seen in SCLL containing sphingosine bovine CER. The SCLLs comprising CER 3 and CER 3B show several coexisting SPPs along with a crystalline CER 3 phase. Moreover, DSC measurements support the destabilizing role of oleic acid (OA) as an SC constituent, especially for phytosphingosine CER SCLLs, as the melting temperatures decreased with the inclusion of OA. The effect of OA on alkyl chain ordering of the lipid matrix in the SCLL variants was supported by FTIR. Finally, a fluorescence-based hydrophilic dye-release assay demonstrates that the SCLL variants responded differently to subsolubilizing concentration of Triton X-100, highlighting the high sensitivity of membrane permeability to chemical configurations of CERs and FFAs. For example, OA increases the dye leakage of SCLL in general, while the phytosphingosine CER with α-hydroxylated head group and C18:0 chain results in an increasing resistance to Triton X-100.

We plan to use SCLL as an in vitro model system for screening of pharmaceutical or cosmetic actives that enables rapid evaluation of their effect on membrane properties [49]. In a longer perspective, we envision to translate the molecular insights obtained herein into a design template for SCLL model membrane system that mimics the various SC phase behavior associated with different physiological states.

## 2. Materials and Methods

### 2.1. Materials

Nonhydroxy fatty acid CER from bovine brain (trans-D-erythro-2-amino-4-octadecene-1,3-diol) was purchased from Sigma-Aldrich Inc, St. Louis, MO, USA. The FFAs (palmitic acid, oleic acid, stearic Acid), cholesterol, cholesterol sulfate, calcein, and Triton X were purchased from Sigma-Aldrich as well. Other phytosphingosine-ceramides (ceramide 3, ceramide 3B, and ceramide 6) were bought from Sigma-Aldrich. The nucleoporin Whatman Track-Etch Membrane filters were bought from Cytiva Corp., Marlborough, MA, USA. In addition, the Biotech CE Tubing of MWCO: 300 kDa for dialysis was obtained from Thermo Fisher Scientific, Waltham, MA, USA. The capillaries used for SAXS of length 80 mm, outer diameter of 1.5 mm, and wall thickness of 0.01 mm were purchased from Hampton Research Corp., Aliso Viejo, CA, USA. The Calcein and Triton X100 (Sigma-Aldrich Inc, St. Louis, MO, USA) used were mixed with PBS or diluted to different concentrations for experiments. Phosphate-buffered saline (PBS), pH 7.4, was bought from Missui Pharmaceutical Co. Ltd., Tokyo, Japan. Water used in all the experiments were of Millipore standards provided by Milli-Q water filtration system (Merck, Darmstadt, Germany). All organic solvents were purchased from Sigma-Aldrich.

### 2.2. Preparation of Model Stratum Corneum Lipid Liposome (SCLL)

The SCLL liposomes were prepared according to the method reported by Wertz et al. [47]. Briefly, individual lipids were dissolved in chloroform-methanol (2:1 by volume), and appropriate volumes were combined to obtain the corresponding mixture (CERs, cholesterol, palmitic acid, and cholesterol sulfate = 40, 25, 25, and 10 (mole percent), respectively). The lipid mixture was then transferred in a round bottom flask, and the solvent was removed with a rotary evaporator under high vacuum at room temperature. The RBF with the resulting film was then further dried in the desiccator under vacuum for another 1 h. A total of 1 mL phosphate-buffered saline (10 mM PBS) at pH 7.2 was added to the dried lipid thin film to make a 25 mM lipid suspension. For calcein-encapsulated liposomes, 1 mL of 30 mM calcein in PBS was used. The lipid suspension was occasionally shaking at 80 °C for 2 h, and multilamellar vesicle would form. The resultant multilamellar vesicles were extruded by passing through a 200 nm PC membrane filter (Avanti Polar Lipid Inc., Alabaster, AL, USA) at 80 °C 21 times using a 1 mL a miniextruder (Avanti Polar Lipid Inc., USA) to obtain monodispersed large unilamellar vesicles (LUV). Unencapsulated calcein was removed by overnight dialysis using 300 kDa regenerated cellulose dialysis membrane against PBS (Biotech CE tubing, Spectrum Laboratories, Inc., Rancho Dominguez, CA, USA).

### 2.3. Particle Size and a Polydispersity Index of SCLL

The mean vesicle size and polydispersity index of liposome suspensions were determined using Malvern Zetasizer Nano ZS (Malvern, UK) based on dynamic light scattering. The vesicles were diluted 20 times with PBS in order to avoid multiscattering phenomena, and then they were incubated at 25 °C. Measurements were made in standard disposable cuvettes, (Eppendorf^®^ UVette, Sigma-Aldrich, Singapore), and an average of 30 runs (10 s per run) was collected using the 173° detector. Data were plotted in Microsoft Excel (Microsoft, Washington, DC, USA).

### 2.4. Calcein Leakage Assay

Dialyzed calcein-encapsulated liposomes were 600-fold diluted with PBS before performing the leakage experiment. A total of 245 μL of the diluted calcein-encapsulated liposomes was added to a 96-well flat Black plate (Corning Incorporated, Corning, NY, USA). Fluorescence was measured at 520 nm (excitation at 480 nm) using a fluorescence plate reader (Infinite 200 Pro, Tecan, Salzburg, Austria). A total of 5 μL of 0.02% Triton X-100 (Tx) was added for each well. % of calcein release was calculated based on the following equation: 100 × (*F_t_ − F*_0_)/(*F*_T_ − *F*_0_) %, where *F*_0_ is the initial fluorescence of calcein, *F_t_* is the fluorescence of calcein with the addition of 0.004 wt.% of Tx, and *F*_T_ represents total fluorescence, i.e., complete release of calcein upon addition of 0.2 wt.% of Tx. Fluorescence value was taken for about 20 min with 1 min interval time.

### 2.5. Sample Preparation for Differential Scanning Calorimetry (DSC) Experiment

DSC is widely used for the characterization of lipid phase behavior. It gives the first information about phase changes in the lipid molecules. To prepare the DSC samples, the lipid concentration used 25 mM in PBS buffer. Liposome suspension (20 µL) was carefully placed and sealed in the aluminum hermetic pans. The DSC measurements were performed using a DSC Q10 (TA instruments) with a scanning rate of 5 °C/min. The samples were heated from 10 to 140 °C with an empty aluminum hermetic pan as a reference. 

### 2.6. Sample Preparation for Small-Angle X-ray Scattering (SAXS)

SAXS was used to determine the lipid lamellar organization in the SCLL. The scattering intensity I (in arbitrary units) was measured as a function of the scattering vector Q (Å^−1^). The lamellar repeat distance *d* (lipid bilayer with the adjacent water layer) can be calculated using the relation $d=\frac{2\pi }{Q}=\frac{1}{s}$, where *Q* is the scattering vector connected with the scattering angle 2θ by the equation $Q=\left(\frac{4\pi }{\lambda }\right)sin\theta$. *λ* is the wavelength. From the positions of a series of equidistant peaks (*Qn*), the periodicity or d-spacing of a lamellar phase was calculated using the equation $d=\frac{2n\pi }{Qn}$, with *n* being the order number of the diffraction peak. One-dimensional intensity profiles were obtained by transformation of the 2D SAXD detector pattern from Cartesian (x,y) to polar (ρ,θ) coordinates and subsequently integrated over θ. The X-ray wavelength (Cu-K) was 1.5406 Å. All measurements were performed using Nanoinxider instrument by Xenocs. Data analysis was performed using FOXTROT software version 3.3.4 (Xenocs SAS, Grenoble, France; Soleil, Saint-Aubin, France). For all SAXS measurements, multilamellar SC liposomes were frozen and thawed for several cycles using liquid nitrogen and at 80 °C water bath. They were kept in the water bath for 4–5 min and in vortexes between the freeze–thaw cycles. After hydration, the samples were transferred into borosilicate capillaries of 1 mm diameter and 0.01 mm of wall thickness, which were sealed and stored at room temperature for 1 day.

### 2.7. Sample Preparation for FT-IR Measurement

To obtain information on the lateral organization and conformational ordering of the lipids, FTIR spectra were recorded. The liposome samples were prepared as described above with minor modifications. Briefly, 10 mg lipid mixture was dried under N_2_ flow and then rehydrated with PBS buffer containing D_2_O at 80 °C (final concentration of 25 mM). VERTEX 70, FT-IR Spectrometer from Bruker was used to record spectra at room temperature. Prior to the analysis of each sample, a blank background was collected. PBS buffer prepared in D_2_O was used as a reference. Before measurements, the ATR crystal was cleaned with a Kim wipe wetted with ethanol. A total of 50 µL of SCLL was then placed on the Zn-Se ATR crystal. Each spectrum represented an average of 126 scans. The spectral resolution was 2 cm^−1^. The instrument was continuously purged with dry N_2_. The detector was cooled by liquid nitrogen. Data were collected in absorbance mode. OPUS 6.5 spectroscopy software (Bruker, Billerica, MA, USA) was used to analyze the IR spectra and determine the position of CH_2_ stretching modes. 

## 3. Results and Discussion

### 3.1. Preparation of Multilamellar and Unilamellar SCLLs Model Membranes

Multilamellar and unilamellar SCLLs were prepared and used for different measurements. For SAXS, FTIR spectroscopy, and DSC measurements, multilamellar SCLLs were prepared from rehydration of dried mixtures of lipids without extrusion. For DLS and permeability assays, they were subjected to mechanical extrusion to obtain unilamellar SCLLs. More details are included in the respective section below.

### 3.2. Lamellar Organization of SCLL Model Membranes

In this section, we examine if the selected SC-mimicking lipid compositions self-assemble into the LPP or SPP phases using small-angle X-ray scattering (SAXS) measurement on unextruded, freeze-thawed, and rehydrated samples. Without extrusion, the repeating, concentric bilayer and water layer within the multilamellar vesicles give rise to intense Bragg reflections, which are otherwise masked in the diffuse scattering for extruded, unilamellar samples. Lamellar repeat distances that correspond to the reflections are calculated to determine the existence of LPP or SPP phases. The lamellar phases observed in the different SCLLs are summarized and compared with findings from previous works [27,31,52,53,54,55,56,57,58] in Table S1.

#### 3.2.1. Phytosphingosines CERs and FFAs

**Figure 2:**
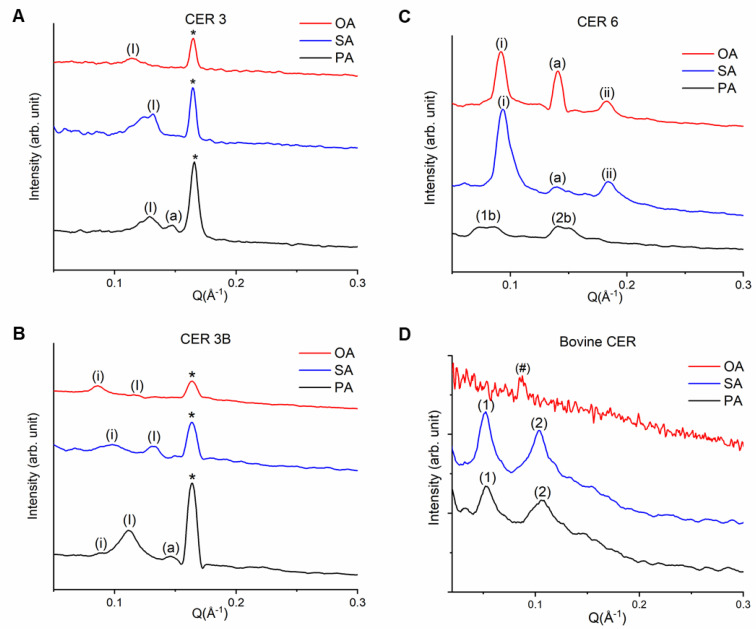
SAXS diffraction profiles of SCLLs. (**A**) CER3-FFAs. (**B**) CER3B-FFAs. (**C**) CER6-FFAs. (**D**) Bovine CER-FFAs. Roman numeral mark “(I)” denotes the diffraction peaks attributed to SPP (48.3 Å–57.1 Å). * Represents crystalline phase of CER 3 (39.3 Å). “(a)” denotes additional SPP (41.9 Å–44.9 Å). “(i)” and “(ii)” represent 1st- and 2nd-order diffraction order of phase with repeat distance 62.8 Å–69.8 Å. “(1b)” and “(2b)” represent 1st- and 2nd-order diffraction of 78.6 Å–83.8 Å phase. The peaks “(1)” and “(2)” indicate the 1st- and 2nd-order diffraction of the LPP (125.7 Å). The LPP phase for Bovine-CER-OA with periodicity 139.6 Å is indicated by the hash sign “(#)”.

CER3-FFA models exhibit SPPs with Q values 0.13 Å^−1^ (PA), 0.13 Å^−1^ (SA), and 0.11 Å^−1^ (OA) that correspond to repeat distances of 48.3 Å (PA), 48.3 Å (SA), and 57.1 Å (OA), respectively (Figure 2A, labeled by “(I)”), consistent with reported values [5,8,59]. In addition, the sharp peak present at 0.16 Å^−1^ (denoted by “*”) reveals the presence of a 39.3 Å phase that is attributed to crystalline CER 3 [13]. For CER3-PA, an additional phase appears with a repeat distance of 41.9 Å (denoted by letter “(a)”). The larger repeat distance for CER3-OA (57.1 Å) is likely attributed to an increase in the interlamellar water layer thickness due to fluctuation of the bilayer in the midplane region arising from a mismatch of acyl chain conformation of the two constituents [37,60]. The CER3B-FFA models exhibit SPPs with multiple repeat distances. Two different phases are evident for CER3B-PA, with characteristic Q values of 0.09 Å^−1^ (denoted by “(i)”) and 0.11 Å^−1^ (denoted by “(I)”), that correspond to repeat distances of 69.8 Å and 57.1 Å, respectively (Figure 2B, black line). CER3B-SA shows two phases with Q values of 0.10 Å^−1^ (denoted by “(i)”) and 0.13 Å^−1^ (denoted by “(I)”), with repeat distances of 62.8 Å and 48.3 Å, respectively (Figure 2B, blue line). The OA-containing membrane displays two phases with peaks positioned at Q = 0.09 Å^−1^ (denoted by “(i)”) and 0.12 Å^−1^ (denoted by “(I)”), with repeat distances of 69.8 and 52.4 Å, respectively (Figure 2B, red line). For the three CER3B-FFA SCLLs, a crystalline CER 3B phase is also detected (denoted by “*”). For CER3B-PA, an additional phase appears with a Q value of 0.15 Å^−1^, which corresponds to a repeat distance of 41.9 Å (denoted by letter “(a)”). In comparison to CER 3 SCLLs, larger repeat distances (denoted by “(i)”) are present in the CER 3B SCLLs. Moreover, the presence of OA into CER 3B SCLL further expands the SPP phase as compared to SA-containing CER 3B SCLLs. These observations are consistent with a disordering effect of the unsaturated fatty acyl chains [37,60] and weakly supported by occasional, small upward shifts of both the asymmetric and symmetric CH_2_ stretching vibrations in the FTIR spectra (Figure S1). 

The CER 6-PA SCLL sample displays two broad diffraction features at Q = 0.08 and 0.15 Å^−1^ (denoted by “(1b)” and “(2b)” for 1st- and 2nd-order peaks, respectively), with repeat distances falling in the range of 78.6 Å–83.8 Å (Figure 2C, black line). In the case of CER 6-SA and CER 6-OA, the two peaks at Q = 0.09 and 0.18 Å^−1^ are attributed to a lamellar phase with a repeat distance of 69.8 Å (denoted by “(i)” and “(ii)” for 1st- and 2nd-order peaks, respectively) (Figure 2C, blue and red line), which is in good agreement with data by Bouwstra et al. [18]. In addition, unknown phases at 0.14 Å^−1^ (denoted by “(a)”), attributed to the lamellar repeat distance of 44.9 Å are identified for CER 6-SA and CER 6-OA. In contrast to the CER 3- and CER 3B-containing SCLLs, it is notable that a crystalline CER 6 phase is not present. Furthermore, it is noted that the presence of a longer FFA in CER 6-containing SCLLs reduces the repeat distance for the main phase, as compared to PA-containing CER 6 SCLLs, whereas monounsaturation of the FFA does not alter the repeat distance. This differential behavior of OA on CER 6-containing SCLL, as compared to that on CER 3- and CER 3B-containing SCLLs, is likely associated with the presence of the alpha-hydroxy fatty acid moiety, which generally promotes better miscibility [39] by forming stronger in-plane hydrogen bonding with adjacent lipids [38,61].

#### 3.2.2. Sphingosine CER and FFAs

Bovine-CER-PA and Bovine-CER-SA show two orders of diffraction peaks “(1)” and “(2)” with a periodicity of 125.7 Å (Figure 2D, black and blue line). In the case of bovine-CER-OA, a less distinct reflection was detected (Figure 2D, red line denoted by “#”), which likely corresponds to an increased periodicity of 139.6 Å. This suggests that a disordering effect of the unsaturated fatty acyl chains is in play [10,37,42,60], which is qualitatively supported with the increase in the asymmetric stretching frequency with changing FFA from saturated SA (C18) to unsaturated OA (C18) in the FTIR spectra (Figure S1). The formation of the LPP phase, instead of SPP, as observed for the three phytosphingosines SCLLs, is likely driven by the presence of the sphingosine and a long chain unsaturated nervonic acid moiety in bovine CER [18,62].

Overall, the SAXS analysis indicates that SCLLs specifically mimic the SPP or LPP phases that are ubiquitous in human skin. SCLL model membranes composed of synthetic phytosphingosine CERs (CER 3, CER 3B, CER 6) mimic SPP of human SC, which exhibit diffraction peaks corresponding to repeat distances of about 42–84 Å [5,8]. The LPP phase is detected only for bovine CER-based SCLLs with repeat distances falling in the range 126–140 Å, consistent with the phase preference of sphingosine and phytosphingosine CERs reported earlier [27]. Furthermore, the systematic library of SCLLs that consists of one CER species and one FFA species highlights the existence of domain heterogeneity in the SPP phase, even in such simplified model membranes [63]. 

### 3.3. Thermal Profiles of SCLL Model Membranes

**Figure 3:**
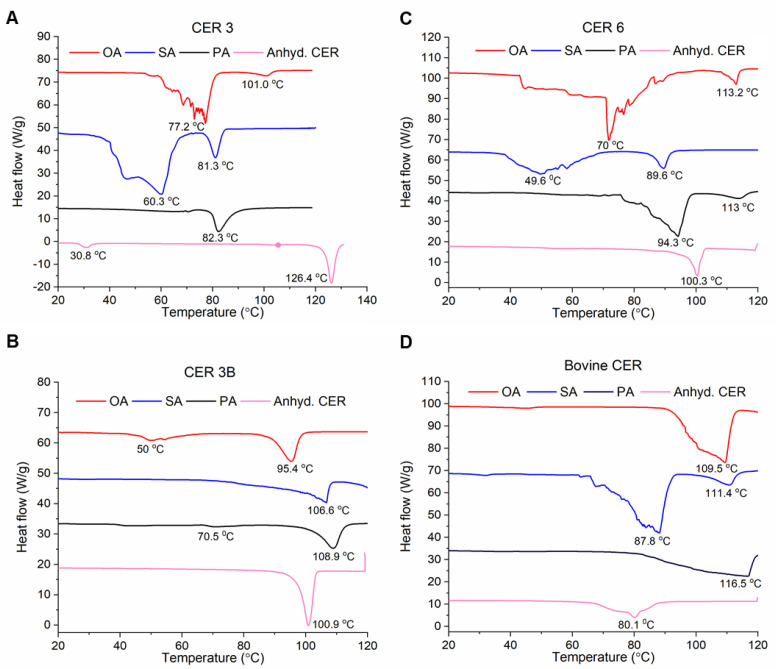
The DSC thermograms of the SCLLs recorded in the heating cycle. (**A**) CER3-FFAs. (**B**) CER3B-FFAs. (**C**) CER6-FFAs. (**D**) Bovine CER-FFAs.

To examine the thermotropic behavior of the lipid organization of the SCLLs, we carried out differential scanning calorimetry (DSC) measurements to determine the thermal phase properties in the range of 10–140 °C obtained at a heating rate of 5 °C/min (Figure 3). Briefly, a sample and a reference are exposed to the same heating regime and the difference in the absorbed (endothermic reaction) or released (exothermic reaction) energy is measured as a function of temperature [64]. For instance, human SC is characterized by multiple endothermic temperature transitions [65,66,67,68]. A phase transition is mostly determined by the extrapolated onset of a DSC peak, and the largest peak in a thermogram typically represents the cooperative melting of hydrocarbon chains. Additional phase transitions, such as the transition from one crystalline packing to another, or structural changes in the polar headgroup region, may also be recorded in the thermal profile. Notably, a thermogram for SCLL is characterized by two major transitions, which include a transition from an orthorhombic to a hexagonal lattice (H_I_) in the lamellar gel phase at lower temperature [5,69], followed by a transition into a nonlamellar inverted hexagonal (H_II_) phase at higher temperature [66,67]. Along with these two transitions, up to eight intermediate transitions have been reported and are relatively well-understood [5,26,66,67,69,70]. Overall, the thermal profiles of the 12 SCLLs investigated are generally characterized by a broad transition and a smaller and sharper transition. The broad transition contains more than one component, suggesting compositionally distinct domains and phases, corroborating with the SAXS profiles above (Figure 2). Since the determination of onset temperatures is difficult in some cases because of broad peaks, the peak maxima of the transitions are reported and described below and summarized in Table S2. Furthermore, the integrated area under the peak corresponds to the transition enthalpy (DH). However, the complexities of the transitions (characterized by multiple peaks) hamper the calculation of meaningful enthalpic information.

#### 3.3.1. CER 3

A single transition is evident for CER 3-PA (82.3 °C). In comparison, two transitions are recorded for CER 3-SA (81.3 °C and 60.3 °C) and CER 3-OA (77.2 °C and 101.0 °C). The thermal profile of anhydrous CER 3 displays a sharp phase transition at 126.4 °C along with a small transition at 30.8 °C (Figure 3A, pink line), which is consistent with published values [54]. The broad transitions for the C18 (OA and SA)-containing CER 3 SCLLs are attributed to the orthorhombic-to-hexagonal lattice transition (H_I_) within the lamellar gel phase, followed by the gel-to-liquid crystalline phase transition [65,71]. Similar phase transitions were also reported by Vaddi et al. [72]. For CER 3-SA, the distinct transition that closely followed at 81.3 °C is likely attributed to the transition of lamellar liquid crystalline to the nonlamellar inverted hexagonal (H_II_) phase. The absence of such a distinct transition in the CER 3-OA thermogram is likely due to the destabilizing effect of OA [73], which leads to the reduction in the phase transition temperature, resulting in an overlap with the preceding broad transitions. Intriguingly, CER 3-PA shows only the H_II_ phase transition. High temperature transitions, such as that observed at 101.0 °C for CER 3-OA previously, was attributed to reordering of the polar headgroup [66,67] or the CER crystalline phase. 

#### 3.3.2. CER 3B

The thermograms of CER 3B SCLLs are generally characterized by a transition at temperature above 90 °C (108.9 °C, 106.6 °C, and 95.4 °C for CER 3B-PA, CER 3B-SA, and CER 3B-OA, respectively) (Figure 3B). Additional, but weak, transitions are detected for CER 3B-PA (70.5 °C) and CER 3B-OA (50 °C). The thermal profile of anhydrous CER 3B shows a sharp transition at 100.9 °C. The apparent absence of the orthorhombic-to-hexagonal lattice transition (H_I_) and gel-to-liquid crystalline transition (H_II_) suggests that the unsaturation in CER 3B may perturb the ordered packing arrangement typically seen in SC membrane. The transition at a much higher temperature is likely due to further fluidization of a CER 3B-enriched phase. Interestingly, the effect of OA in reducing the transition temperature of lipid phases is again demonstrated in the fluidizing phase transition [74,75], weakly supported by occasional, small upward shifts of the asymmetric CH_2_ stretching vibrations in the FTIR spectra (Figure S1).

#### 3.3.3. CER 6

The thermograms of CER6-FFA SCLLs are similar to those of CER 3-FFA SCLLs (Figure 3C). A major transition is seen for CER 6-PA (94.3 °C), along with a minor transition (113.0 °C). In comparison, two transitions appear in the thermogram for CER 6-SA (49.6 °C and 89.6 °C) and CER 6-OA (70.0 °C and 113.2 °C). The transitions can be rationalized with similar assignments of packing lattice and phase transitions, as described for CER 3-FFA SCLLs. Comparing CER 3- and CER 6-containing SCLLs with the same FFA, we find that the transition temperatures characterizing the loss of the lamellar phase (liquid crystalline to H_II_ phase) for CER 6-containing SCLLs are higher for CER 6-PA and CER 6-SA, likely due to the stronger in-plane hydrogen bonding at the headgroup region [38,61]. The much lower apparent transition temperature for CER 6-OA (peaking at ~70 °C) may be due to the overlapping of multiple transitions, effectively masking the lamellar-to-H_II_ phase transition.

#### 3.3.4. Bovine CER 

The thermograms of bovine CER-FFA SCLLs (Figure 3D) are similar to those of CER 3-FFA and CER 6-FFA SCLLs, but the transitions were shifted to a higher temperature range, in general. They are 116.5 °C for bovine-CER-PA, 87.8 °C and 111.4 °C for bovine-CER-SA, and 109.5 °C for bovine-CER-OA. The thermal profile of anhydrous bovine CER is characterized by a broad endothermic feature at 80.1 °C. The exception is CER-SA, which shows a broad transition at 87.8 °C. The thermotropic behavior of bovine CER-FFA SCLLs can be attributed to the mixture of saturated (C18:0) and unsaturated (C24:1) long-chain fatty acid moiety in bovine CER, and the presence of lamellar LPP phase (Figure 2D). Moreover, the general fluidizing effect of OA is less clear, as compared to the SCLL containing the phytosphingosine CERs. 

Collectively, the DSC results support the destabilizing role of OA as an SC constituent, in particular for phytosphingosine CER SCLLs. However, the destabilizing effect of OA is not observed for bovine CER SCLLs, mirroring its marginal effect on the membrane integrity/permeability, as revealed by the functional “leakage” assay discussed below. Furthermore, multiple overlapping endothermic transitions are detected, making detailed assignments impossible. Along with the identification of multiple repeat distances in the SAXS measurements, this further supports the presence of multiple domains or phases within these model membranes. 

### 3.4. Mechanical Extrusion and Characterization of SCLLs

For membrane permeability assay, the multilamellar SCLLs were subjected to mechanical extrusion to obtain unilamellar SCLLs. Vesicle resizing and reduction in the multilamellarity of the SCLLs used for SAXS, FTIR, and DSC as described in the preceding sections are required to achieve a comparable effective detergent-to-lipid ratio. Briefly, multilamellar SCLLs were first rehydrated in a self-quenched calcein medium, followed by passing through a 200 nm membrane multiple times and dialyzed to remove unencapsulated calcein. Dynamic light scattering (DLS) measurements indicate that the hydrodynamic diameters, based on intensity distribution, are comparable for all SCLLs, and fall between 120–170 nm (Table 1). The polydispersity indexes (PdIs) vary between 0.01–0.14, confirming the monodispersity of the extruded SCLLs.

### 3.5. Fluorescence Dye Leakage Assay

**Figure 4:**
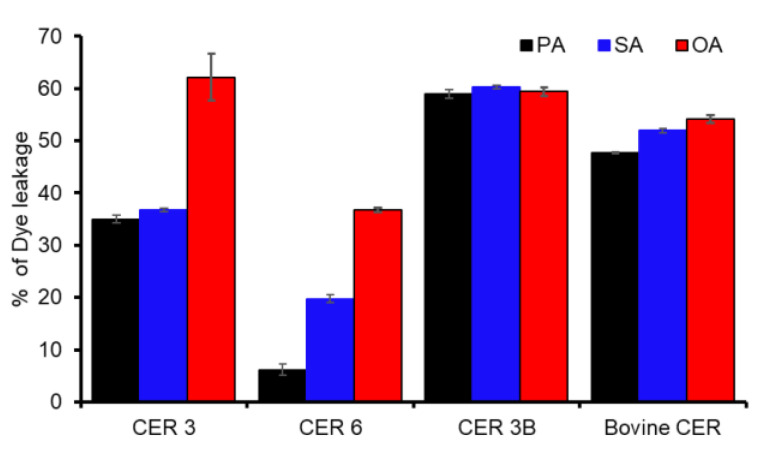
The dye-release study with calcein-loaded SCLLs composed of different CERs and FFAs. Values represent % of calcein release induced by 0.004% Triton X-100 at 40 µM vesicle concentration. The values were taken about 20 min after the addition of Triton X-100. Error bars refer to SEM of three independent experiments.

The primary functional readout of an SCLL is its membrane permeability. As discussed in previous reports, the acyl chain and headgroup configurations of the CERs and FFAs are effective modulators of SCLL membrane permeability [20,26,27]. To assess the effectiveness of CERs and FFAs on membrane permeability, we performed a calcein molecular leakage assay. Briefly, the SCLLs were loaded with calcein, a hydrophilic small-molecule dye, at a self-quenching concentration (30 mM). The release of calcein from its self-quenching environment, across the permeable membrane into the exterior (leakage), results in fluorescence recovery due to dye dilution. The fluorescence intensity is directly dependent on the amount of liberated calcein and correlates to the degree of membrane permeability, represented as percentage of calcein released, as shown in Figure 4. Herein, the SCLLs were subjected to a subsolubilizing concentration of Triton X-100 (0.004%) to examine their susceptibility to detergent.

We first compared the effect of FFAs on the dye release for different CERs. SCLLs comprising OA as the FFA constituent show a distinct increase in fluorescence intensity as compared to their saturated FFA counterparts when combined with CER 3 and CER 6. For bovine CER, only a marginal increase in release is seen, whereas for CER 3B, the release is virtually identical (and at a high level) for all FFAs. When we compare the effect of saturated acyl chain length, CER 6 is noticeably more sensitive to the longer chain FFA, as shown by the significant increase in fluorescence (>10%), between PA- and SA-containing CER 6 SCLLs (Figure 4). Intriguingly, this chain length dependency is marginal for the bovine CER and is not observed for the other CER subtypes. Nevertheless, our results show that CER 6 SCLL exhibits the highest stability against Triton X-100 when combined with PA or SA.

Next, we examined the effect of CER subtypes with varying acyl length/saturation and headgroups. The presence of an unsaturated fatty acid chain in CERs (CER 3B and bovine CER) significantly increases the permeability of the SCLL membrane (Figure 4). Bovine CER-FFAs show a slightly lower leakage when combined with saturated FFAs (PA and SA). On the other hand, the two CERs with saturated acyl chains (CER 3 and CER 6) show a markedly lower fluorescence increase, particularly when mixed with saturated FFAs. The presence of saturated acyl chain moiety in CER, when combined with saturated FFA, gives rise to a stronger tendency to form a highly ordered gel phase with an orthorhombic/hexagonal lattice in the bilayer, as indicated by the thermotropic behavior (Figure 3A,C), which naturally translates into lower permeability [29]. Furthermore, the apparent permeability barrier of CER 6-FFA SCLLs is enhanced by the formation of additional intermolecular hydrogen bonds [26] and the absence of CER crystalline phase with the presence of an α-hydroxylated acyl chain. Hence, the saturated and α-hydroxylated acyl chain of CER 6 endow CER 6 SCLLs the highest stability (lowest permeability) among all the SCLLs evaluated. When comparing SCLLs with the same FFA constituent, CER 6-OA is at least 1.5-fold more stable (less leakage) compared to CER 6-OA and bovine CER-OA, whereas CER 6-PA reaches a maximum of 4-fold when PA-containing SCLLs are compared.

## 4. Discussion and Conclusions

In summary, our study demonstrates that short chain phytosphingosine ceramides with FFAs forms SPPs, whereas long chain (a mixture of C18:0 and C24:1) bovine CER has a tendency to form LPPs similar to those found in human SC. Importantly, the structural organization of SCLL is highly sensitive to small differences in the FFA and CER structures (degree of unsaturation and chain length), especially when comparing membranes containing saturated versus unsaturated fatty acid. We find that the presence of an unsaturated fatty acid such as OA results in more fluidic and disordered membrane, whereas saturated fatty acid and CERs make the membrane more ordered. Moreover, OA lowers the gel-to-liquid crystalline phase transition temperature of SCLL membranes prepared from CERs having saturated acyl tails and has a detrimental effect on the membrane integrity, as revealed by an increasing dye permeability. In addition, membrane permeability of SCLL is controlled partly by CER constituents, such as α-hydroxylation of fatty acyl chain (CER 6) and unsaturated backbones (CER 3B and bovine CER). The nuances in chemical architecture of the SC lipid constituents on functional ramification (membrane permeability) could serve as a guide in designing novel screening tools of skin care molecules and drugs to address their penetration behavior, membrane softening/smoothing effects, and bioavailability. 

Our findings further highlight the high sensitivity of membrane permeability to chemical configurations of CERs and FFAs and suggest several noteworthy insights for the design of SCLL lipid composition. ***First***, the prominent leakage-inducing effect of OA is likely due to its kinked structure (unsaturation), which perturbs the packing of the saturated acyl chain in phytosphingosine backbone of CER 3 and CER 6. This effect diminishes when OA is combined with CER 3B, or when bovine CER as a kinked acyl chain configuration is inherent in these CERs. These findings correlate well with their leakage profile even when combined with saturated FFAs. ***Second***, SCLL formulations with a saturated long chain phytosphingosine CERs (CER 3 and CER 6) exhibit a stronger tendency to form ordered gel phase in the bilayer [29], which translates into lower permeability, as shown above. The permeability can be modulated with the incorporation of different FFAs. ***Third***, α-hydroxylation of the acyl chain of CER (i.e., CER 6) provide a significant stabilizing effect on the membrane permeability, as shown by the 1.5–4-fold increase in stability, due to the formation of additional intermolecular hydrogen bonds [26] and the apparent absence of CER crystalline phase. ***Forth***, FFAs acyl chain length- and unsaturation-dependency are not apparent for CERs that consist of unsaturated acyl chain, therefore suggesting that lipid composition is a critical parameter to consider for molecular leakage assay.

The molecular arrangement of CER, FFA, and cholesterol [76] have been studied extensively and have provided insights into the complex molecular makeup. Several models have been discussed, including the Forslind’s domain mosaic model [77], the single gel phase model [78], and the stacked bilayer model [12]. In light of the current findings, it is imperative and instructive to evaluate them in relation to the earlier proposed models. A notable finding of the current study is that the simplified SCLL exhibits heterogenous phases, which is reminiscent of the domain mosaic model [77], in which membrane permeability is associated with the existence of domain borders. However, we do not find a simple correlation between the permeability and domain boundary, which is indicated by the detection of multiple phases by SAXS and DSC. Furthermore, our data indicate that SCLLs packed in an orthorhombic or hexagonal lattice at physiological temperature range show lower susceptibility to the lamellar perturbing effect of Triton X-100, with a few exceptions, including CER 6-PA that shows the lowest permeability with the absence of a clear-to-hexagonal transition. This discrepancy resonates with the still debatable importance of orthorhombic/hexagonal packing lattice for membrane penetration [79,80]. The daunting task to reconcile the structure–function relationship of a CER-containing membrane requires molecular understanding of the distribution of molecular constituents within the bilayer, including preferential association of FFA and the acyl chain of CER [12] and the asymmetric distribution of cholesterol [76]. Alternatively, comparison of simplified model systems with highly similar molecular makeup can reveal nuances that would have otherwise hidden in the complex matrix of information. 

## Figures and Tables

**Table 1:** DLS measurement of SCLL showing average hydrodynamic diameter (nm) and polydispersity index in parentheses.

Ceramide	PA	SA	OA
CER 3 (C18:0, NP) ^1^	132.3 (0.07)	128.6 (0.04)	122.8 (0.09)
CER 3B (C18:1; cis 9, NP) ^1^	144.7 (0.14)	147.0 (0.14)	166.2 (0.07)
CER 6 (C18:0, AP) ^1^	169.8 (0.11)	134.7 (0.01)	152.6 (0.08)
Bovine CER (NS) ^1^	164.7 (0.01)	162.0 (0.08)	165.0 (0.06)

^1^ CER, FFA, cholesterol, cholesterol sulfate = 40, 25, 25, 10, mol%, respectively.

## Data Availability

The data presented in this study are available within the article and Supplementary Materials.

## Supplementary Materials

The following supporting information can be downloaded at: https://www.mdpi.com/article/10.3390/membranes13020135/s1, Figure S1: The FT-IR spectroscopy analyses of SCLLs; Figure S2: Dynamic light scattering (DLS) measurements of extruded SCLL; Table S1: Comparison of lamellar phases observed for different lipid compositions; Table S2: Phase transitions detected in the DSC measurement of SCLLs.
