# Supplementary material for: Biomimetic Stratum Corneum Liposome Models: Lamellar Organization and Permeability Studies
Source: Membranes (Basel). 2023 Jan 20;13(2):135. doi: 10.3390/membranes13020135 (PMC9962386; doi:10.3390/membranes13020135)
Supplement: Supplementary file 1 [file membranes-13-00135-s001.zip › membranes-2145443-supplementary.pdf]

membranes

*Article*

# SUPPLEMENTARY INFORMATION

# Biomimetic Stratum Corneum Liposome Models: Lamellar Organization and Permeability Studies

**Susmita Roy [1], James C.S. Ho [2], Douglas L.C. Teo [1], Shikhar Gupta [2,*] and Madhavan Nallani [1,3,*]**

[1] Center for Biomimetic Sensor Science, School of Materials Science and Engineering, Nanyang Technological University, Singapore 637553
[2] Procter & Gamble International Operations SA SG Branch, 138547, Singapore
[3] 3ACM Biolabs Pte Ltd, 71 Nanyang Drive, 638075, Singapore
* Correspondence: SG, s.gupta@pg.com; MN, mnallani@acmbiolabs.com

**The supplementary material contains the following:**
**Figures S1-S2**
**Table S1-S2**

**Citation:** Roy, S.; Ho, J.C.S.; Teo, D.L.C.; Gupta, S.; Nallani, M. Biomimetic Stratum Corneum Liposome Models: Lamellar Organization and Permeability Studies. *Membranes* **2023**, *13*, 135. https://doi.org/10.3390/membranes13020135

Academic Editor: Marija Raguž

Received: 22 December 2022
Revised: 18 January 2023
Accepted: 18 January 2023
Published: 20 January 2023

**Copyright:** © 2023 by the authors. Licensee MDPI, Basel, Switzerland. This article is an open access article distributed under the terms and conditions of the Creative Commons Attribution (CC BY) license (https://creativecommons.org/licenses/by/4.0/).

*FT-IR spectroscopy of stratum corneum lipid liposomes (SCLL)*

FTIR spectroscopy was performed to determine the lateral packing and conformational ordering of lipid matrix. The $CH_2$ symmetric and asymmetric stretching frequency of the hydrocarbon chains provides information on the conformational ordering of lipid tails. The low wavenumbers of the $CH_2$ symmetric (~2849 $cm^{-1}$) and asymmetric (~2918 $cm^{-1}$) vibrations indicate the presence of a highly ordered lipid organization (either hexagonal or orthorhombic). An increase in $CH_2$ stretching frequency represents the formation of a liquid disordered phase.

The $CH_2$ stretching vibrations of (both symmetric and asymmetric) CER:FFA: cholesterol:cholesteryl sulfate formulations are plotted in Fig. S1. The result shows that the SCLLs formulated with both saturated CERs (CER 3, CER 6) and FFAs chain have an initial CH2 stretching between 2849.0 to 2850 $cm^{-1}$ (symmetric) and 2917 to 2819 $cm^{-1}$ (asymmetric) respectively. These values of CH2 stretching are characteristic of tightly packed ordered chains conformation. The incorporation of unsaturated FFA (OA) in liposomal membrane increase $CH_2$ stretching frequency values to 2851 $cm^{-1}$ (symmetric) and 2820 $cm^{-1}$ (asymmetric), suggesting unsaturated fatty acid chain turns the lipid bilayer to a more disordered phase. The most remarkable change was observed in the Bovine CER formulation, where asymmetric stretching frequency shifts from 2917 to 2920 $cm^{-1}$ with changing FFA from saturated SA to unsaturated OA. Moreover, in CER 3B formulations, asymmetric stretching frequency increases to 1-2 $cm^{-1}$ compared to CER 3 formulations, for the same FFA. The result indicates that CER 3B makes bilayer more disordered due to the presence of unsaturation in CER acyl backbone. Thus, both CER acyl chain and FFA structure impact the conformation ordering of lipid matrix in SC bilayer.

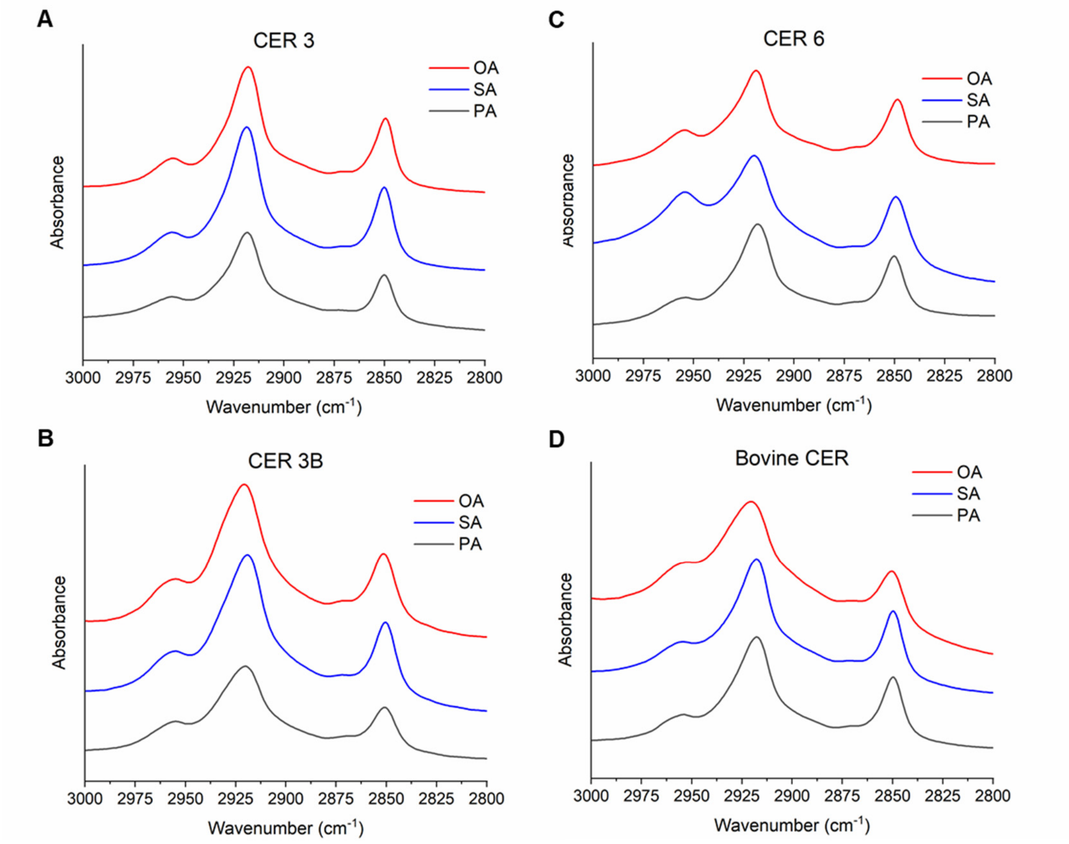


**Figure S1. The FT-IR spectroscopy analyses of SCLLs.** Representative plots of (A) CER3-FFAs. (B) CER3B-FFAs. (C) CER6- FFAs. (D) Bovine CER-FFAs.

*Dynamic light scattering (DLS) measurement of SC lipid liposomes (SCLL)*

The 12 SCLLs were extruded and examined by dynamic light scattering (DLS). The hydrodynamic diameters, based on intensity distribution are comparable for all SCLLs, and fall between 120-170 nm (Fig. S2 and Table 1). The polydispersity indices (PDIs) vary between 0.01-0.14, suggesting that monodisperse SCLLs are formed.

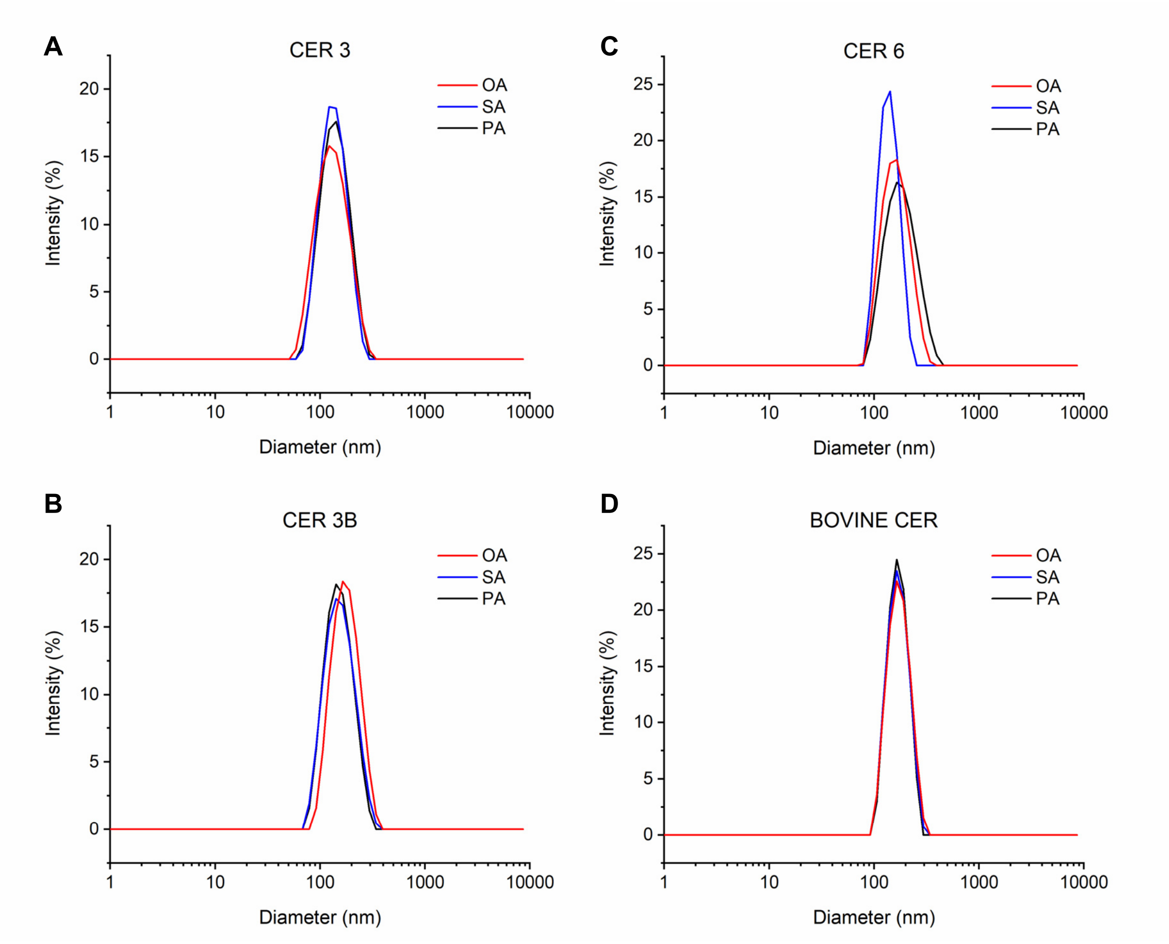


**Figure S2. Dynamic light scattering (DLS) measurements of extruded SCLL.** Intensity-weighted size distributions are shown for (A) CER3-FFAs, (B) CER3B-FFAs, (C) CER6- FFAs, and (D) Bovine CER-FFAs.

Table S1. Comparison of lamellar phases observed for different lipid compositions.

| No. | Lipid composition | SPP (Å) | LPP (Å) | Others (Å) | Ref. |
|---|---|---|---|---|---|
| | CER 3 (NP) | | | | |
| 1. | CER 3:FFA mixture:cholesterol (44:31:25, wt%) FFA mixture = OA:LA:PA:MA:SA (10.7:4.0:11.9:1.2:3.2) | 74.5 | - | - | [54] |
| 2. | CER 3:lignoceric acid:cholesterol (1:1:1, mol%) + 5mol% Cholesteryl Sulfate | 52.4 | - | 42.5, 37.1 | [31] |
| 3. | CER 3:FFA:cholesterol:cholesterol sulfate (40:25:25:10, mol%) | | | | This study |
| | FFA = PA | 41.9, 48.3 | | 39.3 | |
| | FFA = SA | 48.3 | | 39.3 | |
| | FFA = OA | 57.1 | | 39.3 | |
| | CER 6 (AP) | | | | |

| | | | | | |
|---|---|---|---|---|---|
| 4. | CER 6:PA:cholesterol:cholesteryl sulfate (55:15:25:5, mol%) | 46.0 | - | 42.1 | [56] |
| 5. | CER 6:FFA:cholesterol:cholesteryl sulfate (40:25:25:10, mol%) | | | | This study |
| | FFA = PA | 78.6 – 83.8 | - | - | |
| | FFA = SA | 69.8, 44.9 | - | - | |
| | FFA = OA | 68.0, 44.9 | - | - | |
| | Bovine CER (NS) | | | | |
| 6. | Bovine CER:FFA mixture:cholesterol (1:1:1, mol%) FFA mixture = C16:C18:C20:C22:C22:3:C24:C26 (1.3:3.3:6.7:41.7:5.4:36.8:4.7) | 57.6, 51.8 | - | 33.5 | [53] |
| 7. | Bovine CER:lignoceric acid:cholesterol (1:1:1, mol%) + 5mol% cholesterol sulfate | 53.9 | - | 34.1 (c) | [31,52] |
| 8. | Bovine CER(C24):FFA mixture:cholesterol (1:1:1, mol%) FFA mixture = C16:C18:C20:C22:C24 (1.8:4.0:7.6:47.8:38.8, mol%) | 54.0 | 122 | 33.8 (c) | [55] |
| 9. | [CER EOS(C30):Bovine CER(C24)]:FFA mixture: cholesterol ([4:6]:1:1, mol%) FFA mixture = C16:C18:C20:C22:C24 (1.8:4.0:7.6:47.8:38.8, mol%) | - | 125 | 37 | [58] |
| 10. | Bovine CER:Cer EOS:lignoceric acid:cholesterol (0.7:0.3:1:1) + 5 wt% cholesterol sulfate | - | 121.9 | 34 | [57] |
| 11. | [CER 1:CER 3:Bovine CER (AS)]: FFA mixture: cholesterol ([1:7:2]: 1:1) FFA mixture = C16:C18:C20:C22:C22:3:C24:C26 (1.3:3.3:6.7:41.7:5.4:36.8:4.7) | 57.1 | 128 | 37.2 | [27] |
| 12. | Bovine CER:FFA:cholesterol:cholesterol sulfate (40:25:25:10, mol%) | | | | This study |
| | FFA = PA | - | 125.7 | - | |
| | FFA = SA | - | 125.7 | - | |
| | FFA = OA | - | 139.6 | - | |
| | CER 3B | | | | |
| 13 | CER 3B:FFA:cholesterol:cholesterol sulfate (40:25:25:10, mol%) | | | | This study |
| | FFA = PA | 41.9, 57.1, 69.8 | - | 39.3 | |
| | FFA = SA | 48.3, 62.8 | - | 39.3 | |
| | FFA = OA | 52.4, 69.8 | - | 39.3 | |

AS = CERs consisting of an α-hydroxylated (A) fatty acid moiety and a sphingosine (S) moiety.

Table S2: Phase transitions detected in the DSC measurement of SCLLs.

| Ceramides $T_m$ (°C) | PA | | SA | | OA | |
|---|---|---|---|---|---|---|
| | $T_m$ (°C) ΔH (J/gm) | Phase Transition | $T_m$ (°C) ΔH (J/gm) | Phase Transition | $T_m$ (°C) ΔH (J/gm) | Phase Transition |
| CER 3<br><br>$T_m$ (1): 126.4<br>$T_m$ (2): 30.8 | $T_m$ (1): 82.3 | liquid crystalline to $H_{II}$ phase | $T_m$ (1): 60.3 | Orthorhombic to hexagonal I packing to $H_{II}$ phase | $T_m$ (1): 77.2 | orthorhombic→ hexagonal → liquid crystalline → $H_{II}$ phase |
| | | | $T_m$ (2): 81.3 | gel → liquid crystalline→ $H_{II}$ phase | $T_m$ (2): 101.0 | Further fluidizing transition |
| CER 3B<br>$T_m$: 100.9 | $T_m$ (1): 70.5 | Orthorhombic to hexagonal packing to hexagonal II phase | $T_m$ (1): 106.6 | Further fluidizing transition | $T_m$ (1): 50 | orthorhombic to hexagonal packing |
| | $T_m$ (2): 108.9 | Further fluidizing transition | | | $T_m$ (2): 95.4 | Further fluidizing transition |
| CER 6<br>$T_m$: 100.3 | $T_m$ (1): 94. 3 | liquid crystalline to $H_{II}$ phase | $T_m$ (1): 49.6 | orthorhombic to hexagonal packing | $T_m$ (1): 70.0 | orthorhombic→ hexagonal → liquid crystalline → $H_{II}$ phase |
| | $T_m$ (2): 113.0 | Further fluidizing transition | $T_m$ (2): 89.6 | gel → liquid crystalline → $H_{II}$ phase | $T_m$ (2): 113.2 | Further fluidizing transition |
| Bovine CER<br>$T_m$: 80.1 | $T_m$ (1): 116.5 | fluidizing transition | $T_m$ (1): 87.8 | Orthorhombic to hexagonal I packing to hexagonal II phase | $T_m$ (1): 109.5 | fluidizing transition |
| | | | $T_m$ (2): 111.4 | Further fluidizing transition | | |
